# Herbal and Spice Additives in Functional Confectionery Products: A Review

**DOI:** 10.3390/molecules30163449

**Published:** 2025-08-21

**Authors:** Savelii Ishchenko, Urszula Złotek

**Affiliations:** Department of Biochemistry and Food Chemistry, University of Life Sciences in Lublin, Skromna Str. 8, 20-704 Lublin, Poland; savelii.ishchenko@up.lublin.pl

**Keywords:** confectionery product, herbs and spices, bioactive compounds, functional food, food fortification

## Abstract

Herbs and spices are a rich source of bioactive compounds that exhibit multidirectional health-promoting effects. Their bioactivity is mainly related to the presence of phenolic acids, flavonoids, carotenoids, essential oils, alkaloids, tannins, saponins and other plant bioactive compounds. The potential of herbs and spices in preventing oxidative stress, inflammation, bacterial infections and metabolic disorders is increasingly highlighted in the scientific literature, making them a valuable addition to functional foods. Confectionery products belong to a group of food products characterised by high consumer acceptance due to their attractive sensory qualities. Unfortunately, despite high popularity, traditional confectionery mainly provides empty calories in the form of simple sugars and saturated fats while lacking valuable nutrients such as vitamins, minerals or bioactive compounds. This review focuses on the composition and bioactive properties of selected herbs and spices, presenting current knowledge on their potential use in the production of functional confectionery products.

## 1. Introduction

Confectionery products belong to a group of food products characterised by high consumer acceptance, due to their attractive sensory qualities—sweet taste, pleasant aroma and aesthetic appearance. Taking into account their composition and production methods, confectionery products can generally be divided into several categories: confectionery (candies, caramels and others), chocolate and chocolate products, flour confectionery (cookies, cookies, crackers, wafers and others) and dairy confectionery (ice cream). Unfortunately, despite high popularity, traditional confectionery mainly provides empty calories in the form of simple sugars and saturated fats while lacking valuable nutrients such as vitamins, minerals or bioactive compounds [1,2]. With the growing nutritional awareness of the public and the increased demand for health-promoting foods, there is increasing pressure to develop modern confectionery product formulations with increased functional value [3,4,5,6].

Functional foods are a category of products that, in addition to their basic nutritional value, show a beneficial effect on one or more bodily functions, improving health or reducing the risk of developing chronic diseases [5,6]. In this context, of particular interest are raw materials of plant origin, including herbs and spices, which show antioxidant, anti-inflammatory, antimicrobial and antihyperglycaemic properties due to the presence of numerous secondary metabolites, such as polyphenols, flavonoids, essential oils or saponins. These compounds can support the body’s defence mechanisms, neutralise free radicals and modulate inflammatory and glycaemic responses [7,8,9].

Herbs and spices have been used for centuries, both in traditional medicine and in the cuisine of many world cultures [8,10]. Their natural origin and positive associations with preventive health care make them an attractive ingredient for food enrichment, which is also true in the context of confectionery products. The incorporation of herbal or spice extracts, plant powders or isolated bioactive compounds into confectionery formulas offers the possibility to create products with a dual function—satisfying sensory needs and supporting consumer health. The implementation of such additives is associated with technological difficulties. Emphasis should be placed on the compatibility of ingredients, the thermal and oxidative stability of active substances, and the impact on the organoleptic properties of the final product [11,12].

Studies indicate that the use of microencapsulation techniques, freeze-drying and suitable carriers can significantly improve the stability and bioavailability of herbal or spice bioactive ingredients while maintaining attractive sensory properties. Of particular interest are confectionery-based supplement forms—such as candies, lozenges, jellies or bars. These can act as a convenient carrier of health-promoting substances, especially for children, the elderly and consumers avoiding classic tablets or capsules [13,14].

**Figure 1:**
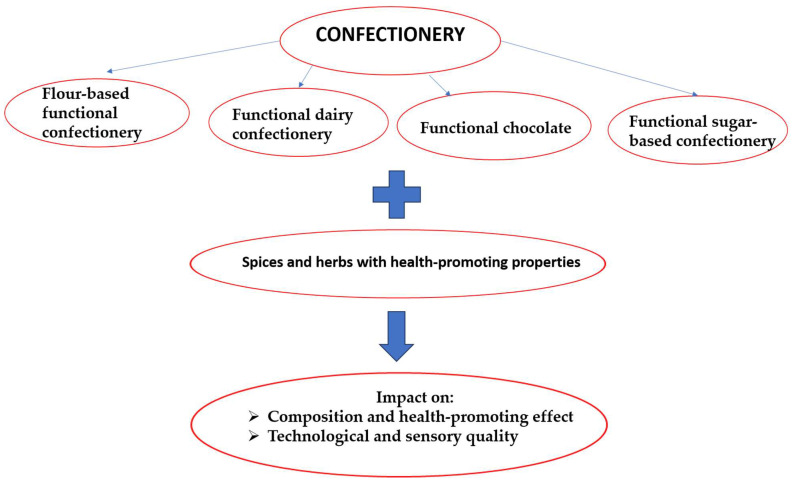
The main aspects discussed in the article.

This review article contains the bioactive properties of selected herbs and spices (especially those used in the production of functional foods) and their mechanisms of action. Additionally, the paper reviews functional confectionery products, and summarizes and discusses the impact of fortifying these products with herbs and spices on their health-promoting properties and technological and sensory quality. Finally, it discusses the current opportunities and limitations of their use in the production of functional confectionery products. It is summarised in Figure 1.

## 2. Bioactive Properties of Selected Herbs and Spices

Herbs and spices are a rich source of bioactive compounds that exhibit multidirectional health-promoting effects. Their bioactivity is mainly related to the presence of phenolic acids, flavonoids, carotenoids, essential oils, alkaloids, tannins, saponins and other secondary metabolites. The potential of herbs and spices in preventing oxidative stress, inflammation, bacterial infections and metabolic disorders is increasingly highlighted in the scientific literature (Table 1), making them a valuable addition to functional foods [15,16,17,18,19]. Table 1 lists the most important spices/herbs used in food fortification.

*Salvia officinalis* L. (sage) is a perennial herb widely used in traditional medicine and culinary practices. Sage contains significant amounts of rosmarinic acid, carnosol, luteolin and thujone [20,21]. In vitro studies have shown strong antioxidant and antimicrobial properties of sage extracts, especially against strains of *Staphylococcus aureus*, *Escherichia coli* and *Listeria monocytogenes*, which are commonly involved in food spoilage and foodborne illnesses. Furthermore, phenolic compounds present in sage may affect signalling pathways associated with inflammatory processes (e.g., pro-inflammatory transcription factor NF-κB), confirming its anti-inflammatory potential [21,22,23].

*Rosmarinus officinalis* L. (rosemary) is a source of compounds such as carnosic acid, carnosol, rosmarinic acid and eugenol. These compounds show high antioxidant activity and the ability to chelate transition metal ions, which can protect lipids from peroxidation in food products [24,25]. In addition, rosemary extracts show antimicrobial activity against a wide range of food spoilage-causing microorganisms and foodborne pathogens. Some compounds found in this herb have been linked to the modulation of cytokine expression and protection of neuronal cells against oxidative stress. Rosemary’s anti-inflammatory and neuroprotective effects also make it a good candidate for functional foods as it targets chronic inflammatory states and cognitive health [26,27].

*Mentha piperita* L. (peppermint) is a widely used medicinal and culinary herb. The main active constituents of mint are menthol, menthone, hesperidin and rosemary acid. Mint is characterised by its spasmolytic, and anti-inflammatory effects. Furthermore, its essential oils exhibit strong antimicrobial activity. Thanks to its strong aromatic properties and cooling effect, mint oil is readily used in confectionery products, not only for its sensory qualities, but also for its functional properties [28,29,30].

*Thymus vulgaris* L. (thyme) is an herbaceous plant from the *Lamiaceae* family traditionally used for its sensory and health promoting properties. The active compounds contained in thyme are mainly thymol and carvacrol, which belong to the monoterpene group. Studies confirm that thyme extracts exhibit strong antimicrobial activity, especially against Gram-positive pathogenic bacteria. Thyme also exhibits anti-inflammatory and antioxidant effects, primarily attributed to its flavonoids and phenolic acids. In addition, thyme has beneficial effects on the respiratory and digestive systems, and its bioactive components can act as natural preservatives in food products enhancing product safety and extending shelf life without synthetic additives [31,32,33,34,35,36].

*Lavandula angustifolia* L. (lavender) is an herbaceous plant known for its relaxing aroma and diverse biological activities. Rich in linalool and linalyl acetate, lavender exhibits sedative, anti-anxiety and antimicrobial properties. Due to its delicate, pleasant aroma and beneficial neuroprotective effects, lavender is gaining popularity as an additive in chocolates, relaxation pastilles and other supplemental and confectionery products [37,38,39].

*Curcuma longa* L. (turmeric) is a perennial plant in the *Zingiberaceae* family, grown for its aromatic rhizome. The main bioactive compounds are curcuminoids, primarily curcuminoids, as well as turmerone-containing essential oils. Curcumin exhibits potent anti-inflammatory effects by inhibiting NF-κB and increasing the activity of antioxidant enzymes (e.g., Nrf2), thereby markedly reducing oxidative stress and inflammation. In addition, turmeric extracts show antibacterial, antimicrobial activity against strains responsible for surgical and wound infections, as well as gastrointestinal and food spoilage-causing pathogens. Recent studies also suggest curcumin’s potential for antiviral activity, including against SARS-CoV-2, by inhibiting replication and protecting nerve cells [40,41,42,43,44,45,46].

*Zingiber officinale* L. (ginger) is a perennial plant of the ginger family, prized for its characteristic aroma and traditional use in traditional medicine. The main bioactive compounds are gingerols, shogaols and paradols [47,48]. Studies have shown that ginger exhibits potent anti-inflammatory effects by inhibiting the NF-κB and COX-2 pathways, as confirmed by both in vitro and in vivo results. In addition, its extracts show the ability to neutralise free radicals, which contributes to the reduction of oxidative stress. Recent studies also highlight an immunomodulatory effect, supporting the immune system by regulating the balance of cytokines and antioxidant enzymes. Ginger may support the treatment of inflammation, infections and metabolic diseases [47,49,50,51,52].

*Ocimum tenuiflorum* L., commonly known as holy basil, tulasi or tulsi, is a perennial plant of the *Lamiaceae* family, valued in Ayurvedic medicine (an alternative medicine system). The main bioactive compounds of this herb are eugenol, ursolic acid, carvacrol and rosmarinic acid. Studies have shown that tulsi leaf extracts have potent antibacterial activity, inhibiting the growth of *Staphylococcus aureus*, *Escherichia coli*, Streptococcus spp and Corynebacterium spp., among others [53,54,55]. In addition, studies have confirmed its anti-inflammatory potential. It inhibits the expression of COX-2 and pro-inflammatory cytokines. Experiments on pancreatic cancer cells showed anti-tumour effects by reducing proliferation and inducing apoptosis [56,57].

*Matricaria recutita* L. (chamomile) is an annual plant of the *Asteraceae* family that has been used in herbal medicine for centuries. The main bioactive compounds are flavonoids (apigenin, luteolin), essential oils (α-bisabolol, chamazulene) and phenolic acids. Studies have shown strong antioxidant and antibacterial effects of methanolic and ethanolic extracts, especially against *Staphylococcus aureus* and *Escherichia coli* [58,59]. Chamomile extracts and essential oils are also effective as natural anti-inflammatory agents, inhibiting the production of NO and inflammatory cytokines in macrophages and T lymphocytes. In addition, immunomodulatory properties have been demonstrated, including inhibition of T lymphocyte activation and reduction of ROS levels, as confirmed by studies with human immune cells. Chamomile may support skin, gastrointestinal and immune system health [60,61,62].

*Ocimum basilicum* L. (common basil) is an annual plant of the *Lamiaceae* family, widely used in cooking and folk medicine. The main bioactive constituents are essential oil compounds—mainly linalool, eugenol and methylchavicol—as well as phenolic compounds, such as rosmarinic acid. Studies have confirmed that basil essential oil has strong antioxidant properties, effectively neutralising free radicals [63]. In addition, basil extracts have antimicrobial activity against pathogens such as *Escherichia coli* and *Staphylococcus aureus* [64]. In animal model studies, it has also been reported to have anti-inflammatory effects by reducing IL-6, TNF-α and NF-κB activity, which makes it relevant both in supplements and as a functional additive in foods [65].

*Allium sativum* L. (garlic) is a persistent plant of the *Amaryllidaceae* family, widely used both in cooking and in folk medicine. Its active substances are mainly allicin and other sulphur compounds (ajoene and alliin), alongside flavonoids and polyphenols. Studies have shown that garlic exhibits strong antioxidant activity by neutralising free radicals and promoting antioxidant enzymes. In addition, garlic extracts have anti-inflammatory effects as they inhibit NF-κB and reduce inflammatory cytokines (TNF-α, IL-6). Garlic also exhibits immunomodulation, stimulating phagocytosis and the activity of NK cells and T cells. In addition, numerous studies demonstrate its antibacterial and antiviral effects, making it a valuable ingredient in functional foods [66,67,68,69,70,71].

*Melissa officinalis* L. (lemon balm) is a perennial plant of the *Lamiaceae* family; it has a lemony leaf aroma and is widely used in phytotherapy. The main bioactive compounds are geranial, neral, citronellal, rosmarinic acid and flavonoids (e.g., quercetin). Studies have shown that lemon balm extracts exhibit strong antioxidant and anti-inflammatory effects, which have been confirmed both in vitro and in vivo [72,73]. In addition, the essential oil shows antimicrobial and antibiofilm properties against foodborne pathogens, such as *Vibrio parahaemolyticus* and *Listeria monocytogenes* [74,75]. Preclinical studies have also confirmed a gastroprotective effect, indicating shielding of the gastric mucosa and improvement on oxidative markers [76].

*Trigonella foenum-graecum* L. (fenugreek) is a legume in the *Fabaceae* family, traditionally used as a spice and for digestive aid. The main bioactive compounds are saponins, phenolic acids, alkaloids (e.g., trigonelline) and fatty acids, such as linoleic and linolenic acids. Studies in animal models have confirmed its anti-inflammatory and anti-rheumatic effects by reducing joint swelling and enzyme markers [77,78,79]. The aforementioned saponins and phenolic acids also show antioxidant and neuroprotective activity, protecting against oxidative stress and neuropathy in an aluminium chloride-induced Alzheimer’s model [80,81]. Fenugreek is therefore a valuable ingredient for functional foods and supplements to support metabolic and neurological health.

*Glycyrrhiza glabra* L. (liquorice) is a plant of the *Fabaceae* family, root of which is used in traditional Chinese and European medicine practices. The main bioactive compounds are glycyrrhizin (a complex triterpenoid) and its metabolite glycyron (glycyrrhizinic acid), as well as flavonoids such as glabrides and licochalcone. Studies indicate that liquorice extracts exhibit potent anti-inflammatory and antioxidant effects, modulating NF-κB pathways and reducing oxidative stress [82,83,84]. In addition, glycyrrhizin has antiviral activity, particularly against hepatitis C and SARS-CoV-2 [85,86]. Studies have also shown antibacterial and hepatoprotective activity [84]. Recent reports also suggest activity against dengue virus (DENV) [87]. Liquorice is a promising raw material for the development of functional products to support immune, hepatic and general health.

*Nigella sativa* L. (nigella) is a plant of the buttercup family (*Ranunculaceae*), grown mainly for its seeds, which are rich in essential oil. The main active compound is thymoquinone (TQ), and there are also alkaloids, saponins and unsaturated fatty acids. Studies have confirmed its strong anti-inflammatory and antioxidant effects, including by modulating Nrf2 and reducing pro-inflammatory cytokines. Nigella also shows antibacterial and antifungal effects, inhibiting the growth of pathogens such as *Escherichia coli*, * Staphylococcus aureus*, *Candida albicans*. Immunomodulatory effects have also been reported, i.e., an increase in NK cell activity and upregulation of IL-10 in animal models. Additionally, TQ shows anticancer potential by inhibiting the proliferation of cancer cell lines [88,89,90,91,92,93,94,95].

*Origanum vulgare* L. (oregano) is a perennial plant in the *Lamiaceae* family, valued for both its aromatic properties and medicinal uses. The main bioactive compounds are monoterpene phenols, primarily carvacrol and thymol, as well as rosmarinic acid, p-cymene and γ-terpinene. Studies have shown that oregano oil has potent antioxidant and antimicrobial activity, effectively neutralising free radicals and inhibiting the growth of pathogens such as *Escherichia coli* and *Staphylococcus aureus* [96,97,98]. Experiments in animal models have also confirmed anti-inflammatory and nephroprotective properties, indicating that it can protect renal tissues from toxin-induced oxidative stress [99]. With these properties, oregano is gaining potential as a natural preservative and functional food ingredient.

*Echinacea purpurea* L. is a perennial herb of the *Asteraceae* family, traditionally used in folk medicine as an immune booster. The main bioactive compounds are alkamides, polysaccharides and phenols such as chicoric acid [100]. In vivo and clinical studies have confirmed its immunomodulatory effects, including increasing lymphocyte and macrophage activity and regulating IL-6, TNF-α and IFN-γ levels. In animal models, antioxidant, anti-inflammatory and nephroprotective effects have been demonstrated, illustrating renal protection against oxidative stress [101,102]. In addition, echinacea extracts contain constituents with antiviral and antibacterial activity, potentially supporting the therapy of respiratory tract infections [103,104].

### Mechanism of Action, Synergistic Potential and Standardisation of Constituents

**Figure 2:**
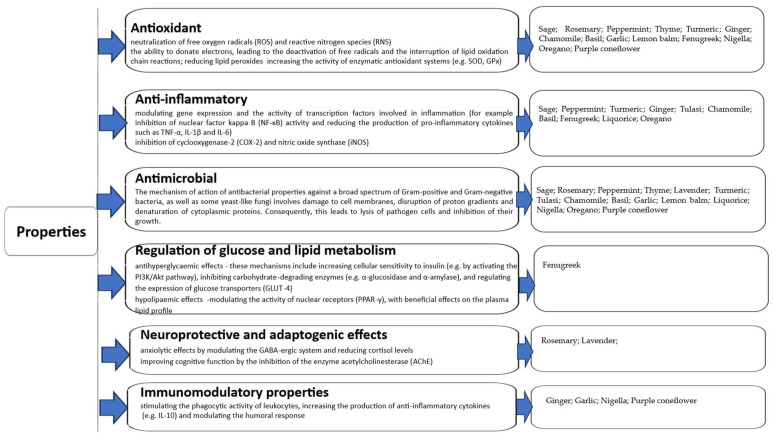
Mechanism of action of selected spices and herbs [108,109,110,111,112,113,114,115,116,117,118,119,120,121,122,123,124,125,126,127,128,129,130,131,132,133,134].

The bioactive compounds in herbs and spices exhibit a variety of mechanisms of action at the molecular, cellular and physiological levels. Their activities include antioxidant, anti-inflammatory, antimicrobial, neuroprotective, hypoglycaemic and immunomodulatory properties (Figure 2).

It is worth noting that the complex chemical composition of herbs often leads to synergistic effects of different groups of compounds. For example, the simultaneous presence of polyphenols and essential oils can enhance antioxidant or antimicrobial activity [105]. Nevertheless, there is a variability in the composition of raw plant material depending on growing, harvesting or processing [106]. To ensure reproducible health effects in functional foods it is necessary to standardise plant extracts [107].

## 3. Functional Food Applications

The development of functional foods is a response to growing consumer interest in products that offer additional health benefits in addition to their nutritional value. Herbs and spices, due to their rich bioactive profile, are a natural source of functional ingredients that can be added to a wide range of food products. The use of herbal or spice extracts, essential oils or powdered plant materials allows food to be enriched in terms of both health-promoting value and sensory attributes.

### 3.1. Herbs in Dairy and Fermented Products

The addition of herbs to dairy products such as yoghurts, kefirs and cheeses has been extensively studied for their antioxidant and antimicrobial potential. Extracts of sage, rosemary and thyme have shown the ability to extend the shelf life of fermented products by reducing the growth of spoilage microflora and protecting against lipid oxidation. An increase in probiotic activity (*Lactobacillus acidophilus*, *Bifidobacterium* spp.) was also observed in studies on yoghurts with peppermint and basil, which may be due to the presence of prebiotic polysaccharides and polyphenols in the plant material [135,136,137].

### 3.2. Herbs in Bakery and Cereal Products

Powdered herbs and their extracts are increasingly used as additives in baked goods, pastries or cereal. The inclusion of thyme, oregano or mint in pastry helps to enrich the flavour profile. Another advantage is the reduction of the glycaemic index of the product and an increase in its ability to neutralise free radicals. Herbs can be used as natural preservatives in bakery products, thus reducing the addition of synthetic chemicals [138,139,140,141].

### 3.3. Herbs in Meat Products and Plant Alternatives

In meat processing technology, plant extracts are mainly used as natural antioxidants and pathogen inhibitors. Rosemary, sage and oregano are effective in reducing myoglobin and lipid oxidation in minced meat, sausages and cured meats. Furthermore, herbs have a beneficial effect on sensory attributes—they impart a characteristic aroma and flavour to products, which increases their consumer acceptance. In the case of vegan and vegetarian products, herbs play a dual role—they improve the taste and enhance the biological value of protein preparations based on soya, peas or plant proteins [139,142,143,144,145].

### 3.4. Herbs in Functional Drinks

Infusions, herbal teas, smoothies and isotonic and energy drinks are another food category in which herbs are widely used. Examples include beverages with lemon balm, lavender and mint, which exhibit relaxing and calming effects. Meanwhile, infusions of rosemary and sage aid in memory and concentration. The introduction of herbal ingredients into beverages often involves standardising extracts and optimising dosage to avoid bitter aftertaste or excessive intensity [146,147,148].

### 3.5. Technological Aspects and Challenges

The use of herbs and spices in functional foods also poses technological challenges. Some of the bioactive substances (especially essential oils and flavonoids) are sensitive to temperature, light and oxygen, which can lead to degradation of the ingredients during heat treatment or storage. In response to these limitations, encapsulation techniques (e.g., microencapsulation, lyophilisation and encapsulation in liposomes) are being developed to increase the stability of bioactive compounds and their controlled release in the gastrointestinal tract [149,150,151,152].

Herbs and spices are increasingly used in various functional food categories due to their health-promoting, antioxidant and antimicrobial properties. However, their introduction into recipes requires consideration of technological and sensory aspects, as well as compliance with food safety regulations and health claims.

## 4. Functional Confectionery Products

### 4.1. Flour-Based Functional Confectionery

In flour-based functional confectionery, the most important ingredient is wheat flour, which has an important role in functional terms and provides volume and structure. The main confectionery products included in this group are cookies, sponge cakes, muffins, and crackers. This confectionery group is characterized by a relatively low nutritional value and a relatively high energy value; therefore, in the creation of functional flour-based products, the main trends focus on increasing the nutritional value, reducing the caloric content and enriching with bioactive ingredients. The challenge for producers of functional confectionery products, such as cookies, which are very popular among consumers, is to create a cookie recipe for diabetics. To this end, attempts are being made to replace some of the white flour in classic recipes with flour with a lower glycaemic index, i.e., wholemeal wheat flour and flours obtained from other cereals and non-cereals with evident nutritive and non-nutritive values, such as oat, barley, rice, soy, buckwheat, flaxseed, etc. An additional strategy is to replace sucrose with lower-calorie alternatives, such as isomalt, maltodextrin, erythritol, xylitol, stevia and steviol glycosides, as well as replacing saturated fats with products containing a lower level of saturated like liquid oils for example rapeseed oil or sunflower oil [153,154]. Additionally, an important aspect in the production of functional products of this group, dedicated in particular to vegetarians and vegans, is the enrichment with protein ingredients, e.g., legume flours or protein powders. An equally important aspect in the production of functional flour-based confectionery products is the fortification of these products with bioactive compounds. Fortification of various types of phyto-additives, most often products of fruit or vegetable origin, such as powders, extracts, or waste products from fruit-vegetable processing, like pomace, results in the creation of products with numerous additional biological properties. It can also contribute to a natural increase in the shelf life of these products and positively influence their organoleptic characteristics [155,156,157,158,159].

### 4.2. Dairy Functional Confectionery

Milk and dairy products are an important part of the human diet due to many nutritious and potential functional ingredients, as well as some precursors of functional compounds [160]. As bioactive constituents occurring in milk, caseins, calcium, lactose, α-lactalbumin, β-lactoglobulin, immunoglobulins, lactoferrin and whey proteins should be mentioned. Milk-based confectionery, for the above reasons, are highly sought-after dessert by consumers. The most popular functional confectionery from this group is ice cream. Ice cream is a sweetened frozen dairy product containing ice crystals, air cells, polysaccharides, proteins and minerals like calcium, sodium and potassium. The functional products market is currently looking for solutions for the production of ice cream, mainly using fortification with bioactive compounds to produce ice cream classified as a functional food. The production process, as well as low storage temperature, predisposes these products to enrichment with both nutritional compounds and, above all, nutraceuticals sensitive to high temperatures. Functional ice creams are most often enriched with insoluble dietary fibre, natural antioxidants, fruit or fruit-derived ingredients or probiotics [160,161].

### 4.3. Functional Chocolate and Chocolate Products

Chocolate is a popular sweet treat enjoyed by people of all ages [162]. Dark chocolate is considered a functional food due to its content of various bioactive compounds, including polyphenols, flavonoids, procyanidins and theobromine, as well as vitamins and minerals. Numerous studies demonstrate the positive impact of dark chocolate on human health, particularly its protective effect against cardiovascular diseases, certain types of cancer and brain-related disorders such as Alzheimer’s disease and Parkinson’s disease, as well as its anti-diabetic, anti-inflammatory and antimicrobial properties. The health-promoting effects of dark chocolate stem from the composition of cocoa, which is an important ingredient in this product. Cocoa beans contain as much as 10% of the dry weight of polyphenols [162,163]. Cocoa beans contain 33–62% cacao butter, which is the main component of dark chocolate. Cocoa butter is composed mainly of triacylglycerols containing 25% palmitic acid, 33% stearic acid and 33% oleic acid [164]. Chocolate production is a multistep process consisting of the harvesting and processing of cocoa beans (fermentation, drying, roasting, nib grinding and refining, conching and tempering). Finally, the content of nutrients as well as bioactive compounds in the final product is lower [165]. Therefore, fortification with nutrients and bioactive ingredients has been used to improve this functional product. The available literature already contains numerous examples of research on chocolate fortification using, among others, fruits, nuts [166,167], spice and herbs [168,169], prebiotic and probiotic [170,171] protein [172], polyunsaturated fatty acids [173,174] and some bioactive components like phenolic compounds [175,176].

### 4.4. Sugar-Based Functional Confectionery

The principal ingredients in sugar confectionery are sucrose, invert sucrose and glucose syrups. Sugar confectionery can be divided into two main groups: boiled sweets (hard candy) and fondant; both consist of sucrose (48%in case of boiled sweets and 62% in fondant), glucose syrup (32% and 16% in boiled sweets and fondant, respectively) and water (20% and 22%, respectively) [177]. Due to the fact that a high sugar content in the diet promotes the development of many diseases, attempts to create functional sugar confectionery products are mainly based on attempts to replace sucrose in the recipes of these products. In this regard, the confectionery industry is looking for new solution to develop low-sugar sweets using natural sweeteners (e.g., stevia glycosides, fruit juices and their concentrates, honey and palm sap sugar) or another sucrose alternatives like sugar alcohols (sorbitol, mannitol, xylitol, maltitol, lactitol and isomalt, etc.) [178,179]. Another direction in creating health-promoting properties of sugar-based confectionery is fortification aimed at increasing the nutritional and/or health-promoting properties of these products because, in their classic form, such candies do not have much nutritional or health-promoting value. Therefore, when creating functional recipes for confectionery products, attempts are made to enrich sweets with products/compounds with a documented positive impact on human health. For this reason, when creating functional confectionery recipes, attempts are made to enrich candies with products/compounds with a documented positive impact on human health, including vitamins, minerals, plant antioxidants, omega-3 fatty acids, probiotics and prebiotics. There are many examples in the available literature of the use of various fruit pulps [180,181], plant extracts [182,183] and by-product waste products [184,185,186,187] for enhancing sugar confectionery with vitamins, minerals, phenolics, fibre and protein. An additional group of sugar-based confectionery products are enriched with compounds such as probiotics [188,189], prebiotics [188], omega-3 fatty acids [190], vitamins and minerals [191] considered medications or dietary supplements [178].

## 5. Confectionery Products Applications

Confectionery products are traditionally characterised by their high content of simple sugars, low nutritional value and limited bioactive ingredients. For this reason, their excessive consumption is considered a risk factor for many diseases of civilisation, such as obesity, type 2 diabetes and cardiovascular diseases. Due to the growing interest in healthy lifestyles and the search for foods with beneficial effects on the body, attempts are increasingly being made to enrich confectionery products with bioactive substances of plant origin, including herbs and spices. The most important goal of enriching confectionery products with herbs and spices is to enhance them with compounds with health-promoting properties. However, as numerous studies show, adding herbs or spices can also modify other features of confectionery products, such as nutritional value, technological features or sensory quality of finished products (Table 2).

The incorporation of some herbs/spices modified the chemical composition of confectionery products, including the content of nutrients, i.e., protein, ash, polysaccharides, including dietary fibre, and of course, the content of bioactive compounds. For a long time, there have been two basic trends in the flour-based confectionery industry: improving nutritive value and at same time, reducing the energy value. There are some examples of improving nutritional quality by enriching the product with herbs or spices. In study conducted by Ng et al. [192], butter biscuits fortified with cinnamon powder was characterized by a higher content of protein, ash and dietary fibre, and Aljobair et al. [193] researched clove powder addition to cookies resulted in higher content of ash and some minerals like K, Na, Mg, Fe, P, Zn and Ca. Fortification with herbs/spices can influence also nutritional value of ice cream. So, ginger addition (as juice, paste and syrup) caused decrease in total solids, total soluble solids and fat content, but an increase in ash content in studied ice cream [194]. In turn, in the study by Solanki et al. [195], ice cream enriched with three additives (tulsi paste (2.5%), ginger juice (2.0%) and clove extract (4.0%)) contained fewer total solids and carbohydrate, but more fat, protein and ash content. Also, candies enriched with *Psydrax umbellata* leaf extract were characterized by a higher content of protein, ash, fibre, vitamin C and total minerals [182].

New trends in the functional food industry also concentrate also improving pro-health properties of confectionary. Natural bioactive substances, which are rich in herbs and spices, are largely responsible for shaping these properties. One of the groups of bioactive compounds with widely documented health-promoting properties are phenolic compounds, the content of which has significantly increased in confectionery products fortified with additives such as rosemary, clove, sakura green tea leaves, turmeric and cinnamon, as well as *Psydrax umbellata* [169,182,193,196,197,198,199,200].

The widely studied health-promoting properties of functional confectionery products are their antioxidant properties; hence, there are many examples of modulating these activities by adding herbs or spices. A study of functional chocolate muffin with rosemary extract showed an increased antioxidant activity confirmed by oxidative haemolysis inhibition assay (OxHLIA) compared to control samples [200].

Additionally, rosemary addition to shortcrust cookies improved their antioxidant properties (DPPH method) and resulted in a reduction of acrylamide formation during baking [196].

Similar effects were observed for biscuits and cacao-based bars with thyme and fennel, confirming the possibility of using these herbs as natural antioxidants [201,202].

Clove bud addition to cakes also resulted in improving antioxidant activity measured as ABTS and DPPH radical scavenging capacity as well as reducing power (RP), but effect measured as RP depended on clove bud form added to cakes—namely, the addition of roasted clove bud increased this activity while the addition of raw clove bud did not affect it [197]. Increased antiradical activity against DPPH of confectionery products was observed also in studies using the addition of clove (for enriching cookies), sakura green tea leaves, turmeric, and cinnamon bark (for enriching dark chocolate), as well as *Psydrax umbellate* as an additive to herbal candy [169,182,193,198]. Additionally, fortifying ice cream with ginger and dark chocolate with sakura green tea leaves resulted in an improvement in antioxidant activity, as expressed by the reducing power (RP) of the studied products [194,198].

Antimicrobial activity is very important in the context of safety and shelf life of the final products. Since the ingredients of many herbs and spices have antimicrobial properties, their addition to food products may have a positive impact on the safety and microbiological stability during storage. In the study conducted by Gonçalves et al. [203], the addition of thyme essential oil significantly increased the microbiological stability of the cake, and encapsulation of the added oil further improved this effect. Even after 30 days of storage, cakes with the addition of encapsulated oil in the amount of 0.600 mg/mL were free of yeast and mold contamination (<1 log CFU/g of molds and yeasts), while control cake samples after this time were significantly contaminated with mold (5.21 log CFU/g of molds) [203]. Similarly, in studies by Dev et al. [197], the addition of clove bud to cake increased its antimicrobial properties, and additionally, roasting of clove bud before addition to cake intensified the antimicrobial properties for cake even more. The antimicrobial properties of clove powder added to cookies were also observed in a study conducted by Aljobair [193] where total bacterial, yeast and mold counts were checked. Generally, incorporating clove powder in the cookies increased the antimicrobial properties of cookies during storage. Ginger incorporation improve also microbial quality of ice cream, but type of processed ginger used for fortification was also affected on the microbial activity in ice cream—namely, the best effect showed in ginger syrup incorporated ice cream [194].

By including herbs in confectionery recipes, it is possible not only to improve the health-promoting profile of the product but also to modify its sensory properties. Herbs such as thyme, oregano mint, lemon balm, sage or rosemary can be used in fresh or dried form, as water-alcohol extracts or essential oils [204,205,206].

Generally, as a result of the presence of essential oils and aromatic compounds, herbs and spices influence the taste, aroma and colour of confectionery products. Sensory quality and consumer acceptability of herb/spice enriched confectionery products very often depend on the size and form of the additive. Therefore, research on this aspect is important in the context of the possibility of practical application of research on the enrichment of confectionery products. The addition of cinnamon powder to butter biscuits in the amount of 6% resulted in a lower assessment of all sensory quality parameters (aroma, colour, appearance, crispiness, flavour and overall acceptance), while the 2% addition did not affect the sensory assessment of the tested biscuits [192]. In turn, shortcrust cookies supplementation with freeze-dried aqueous rosemary extracts in amounts of 0.1, 0.2 and 0.5% caused only a slight decrease in overall sensory quality [196]. Slight decrease in sensory quality (especially of shape, crunchiness, and overall acceptability) of cookies fortified with clove powder was also conducted [193]. A decrease in overall acceptability of cake after clove bud powder addition in Dev et al. [197] study was observed, but the scores were also influenced by the amount of addition (the largest decrease for the sample with 0.8 g addition of clove).

The addition of cinnamon to chocolate in the form of powder caused a reduction in some sensory quality parameters, i.e., bitterness, coarseness, thickness, without affecting the assessment of sweetness, cinnamon odour, chocolate odour, and overall acceptability [199]. On the other hand, the sensory assessment of chocolate enriched with cinnamon essential oil depended to a large extent on the amount of the addition [168]. It should be noted that cinnamon bark oleoresin microcapsules added to dark chocolate did not affect the sensory quality of the product [169], which may indicate that this form of addition is very beneficial from the point of view of consumer acceptability.

In the case of ice cream supplemented with lemongrass and curry leaf, the decisive influence on sensory quality had the form of the additive; namely, ice cream with the addition of distillates was rated much better in comparison to ice cream enriched with powder from these raw materials [207]. Considering sensory quality of ice cream, there are some specific properties, such as melt down, that have a particular effect, especially eye appeal, mouth fee, and the first dripping time (the amount of time it takes for a melting ice cream sample to release its first drop of melted liquid). In the study conducted by Pagthinathan [194], ginger addition resulted in decreasing of ice cream melting rate as well as increasing in the value of first dripping time. A similar effect was observed for melt down time in ice cream fortified with tulsi, ginger and clove in Solanki et al. [195].

Candy and lozenges with peppermint and eucalyptus extracts have been shown to refresh and alleviate symptoms of upper respiratory tract infections. Research shows that those extracts are stable in sugar and isomalt matrices after the production process. The final product exhibits antibacterial activity similar to peppermint and eucalyptus, which have traditional uses in various cough and cold drops [208].

Confectionery products enriched with natural plant extracts are often rated higher in terms of originality and perceived ‘wholesomeness’. However, their aromatic intensity must be carefully balanced to avoid overly dominant flavours.

The herbs/spices addition to confectionery products, however, comes with technological challenges. Some phenolic compounds and essential oils can affect dough texture, moisture content, and even baking processes. Additionally, many bioactive substances are heat-sensitive, which can lead to their partial degradation during thermal processing. Therefore, another important aspect of research on the use of herbs and spices in the fortification of confectionery products is research on the technological aspects of the production of such products. Research conducted by Ng et al. [192] showed changes in textural properties of butter biscuits enriched with cinnamon powder; namely, in supplemented biscuits, an increment of firmness and reduction of crispiness was noted. However, the addition of clove powder to cakes caused a decrease in the penetration value during storage compared to the control cakes, which suggests a softer, more tender cake [197]. In turn, the addition of clove powder had a positive effect on the pasting properties (peak viscosity, breakdown, final viscosity and setback) as well as texture properties (hardness, cohesiveness, springiness, adhesiveness and chewiness) of cookies [193]. Dark chocolate enriched with cinnamon bark oleoresin microcapsules was characterized by greater hardness [169], and ice cream enriched with ginger in various forms (ginger juice, ginger paste and ginger syrup) was characterized by higher values of textural parameters, i.e., hardness, springiness, cohesiveness, gumminess and chewiness [194]. The above observations indicate the need for research on technological quality in the design of functional recipes for confectionery products enriched with herbs or spices rich in bioactive ingredients.

**Table 2:** The use of selected herbs and spices in the fortification of confectionery products.

Material Used for Enrichment	Product	Size and Form of the Addition	Impact on Composition and Health-Promoting Effect	Impact on Technological and Sensory Quality	References
Cinnamon	Butter biscuits	Powder (2, 4 and 6%)	Increase protein, ash and dietary fibre contents	Increment of firmness reduction of crispiness Sensory evaluation: 2%—not different 4%—lower scores for aroma and appearance 6%—lower scores for all parameters (aroma, colour, appearance, crispiness, flavour and overall acceptance)	[192]
Rosemary	Shortcrust cookies	Freeze-dried aqueous extracts (0.1, 0.2 and 0.5%)	Increase of total polyphenol content and DPPH radical scavenging capacity (0.5%), Reduction of acrylamide formation	Increment of stickiness (all sizes of addition) and firmness (0.2 and 0.5%) Slight decrease in overall sensory quality	[196]
Thyme	Cake	Free essential oil carried by cereal alcohol (0.125 mg/mL), Essential oil in micro particles carried by cereal alcohol (0.125 mg/mL and 0.600 mg/mL)	Antibacterial activity	Preserving cakes (microencapsulation thyme oil acts 10 times better)	[203]
Clove bud	Cake	Hot plate-roasted, Microwave- roasted and Unroasted clove bud powder (0.4 g, 0.6 g and 0.8 g)	Increase in total polyphenol content increase in antioxidant activity: DPPH and ABTS radical scavenging capacity (all supplemented samples), Reducing power (samples with roasted clove bud addition) antimicrobial properties (increasing in all supplemented cakes)	The penetration value of all clove powder-added cakes decreased during the storage period that suggests a softer, more tender cake Sensory quality: decrease in overall acceptability (the largest for the sample with 0.8 g addition of clove) increasing oxidative stability of cakes	[197]
Clove	Cookies	Clove powder (0.5, 1, 1.5, 2%)	Increment of minerals (K, Na, Mg, Fe, P, Zn, Ca) and ash content Increase of total polyphenol content and DPPH radical scavenging capacity	Improving pasting properties (peak viscosity, breakdown, final viscosity, and setback) improving the texture properties (hardness, cohesiveness, springiness, adhesiveness, and chewiness) Change in colour (decrease of the lightness (L*) and yellowness (b*) values, but increase of the redness (a*) slight decrease in sensory quality (especially of shape, crunchiness, and overall acceptability) Improving the storage stability (the oxidative stability and antimicrobial properties)	[193]
Lemon balm	Chocolate muffins	Extract (2 mg/g)	Increase in antioxidant activity	Inhibition of fungal and bacterial growth. No change in physical parameters, except increased springiness, and decrease in lightness. Preserving quality similar to sorbate	[200]
Oregano	Chocolate muffins	Extract (2 mg/g)	Increase in antioxidant activity	Inhibition of fungal and bacterial growth. No change in physical parameters, except increased springiness, and decrease in lightness. Preserving quality similar to sorbate	[200]
Rosemary	Chocolate muffins	Extract (2 mg/g)	Significant increase in antioxidant activity	Inhibition of fungal and bacterial growth. No change in physical parameters, except increased springiness, and decrease in lightness	[200]
Fennel	Cocoa based bar	essential oil (1%)	Physicochemical and microbiological evaluations demonstrated that the cacao bar enriched with fennel essential oil complies with quality standards	Sensory quality: differences in acceptability depending on the amount of addition: the most acceptable samples with 1% addition	[202]
Sakura green tea leaves	Dark chocolate	Leaves powder (2%)	Increase of total polyphenol content (additionally, a change in proportions of individual classes of phenolic compounds) Increase in antioxidant activity: DPPH and RP	Not analysed	[198]
Turmeric	Dark chocolate	Turmeric powder (8%)	Increase of total polyphenol content (additionally, a change in proportions of individual classes of phenolic compounds) Increase in antioxidant activity: DPPH and RP	Not analysed	[198]
Cinnamon	Dark chocolate	Powder (4.2%)	Enrichment in bioactive compounds, i.e., copaene, cinnamaldehyde, 4-methylene-cyclohexene, bicyclo (3.1.1.heptane, 6-methyl) and bicyclo (3.1. hexan-2-ol, methyl)	Sensory quality: decreasing the evaluation of bitterness, chocolate, coarseness, thickness, and hardness; no effect on sweetness, cinnamon odour, chocolate odour, and overall acceptability	[199]
Cinnamon bark	Dark chocolate	Cinnamon bark oleoresin microcapsules (4, 6 and 8%)	Increase of total phenolic compounds and tocopherols content and DPPH radical scavenging capacity	Sensory quality—without changes Colour: Change in colour (increase of the lightness (L*), yellowness (b*) values, and redness (a*) Texture properties: increasing of hardness	[169]
Cinnamon	Dark chocolate	Essential oil (0.25, 0.50 and 0.75%)	Not analysed	Sensory quality: differences in acceptability depending on the amount of addition: the most acceptable samples with 0.25% addition, the least acceptable with 0.75% addition. Colour: Change in colour (increase of the lightness (L*), but no statistically significant differences in yellowness (b*) values, and redness (a*)	[168]
Lemongrass	Ice cream	Distillate (2.50% and 3.50%) and Leaf powder (0.70 and 0.75%)	Ice creams prepared using lemongrass powder had significantly higher fat, protein, carbohydrates and total solids content when compared to ice creams prepared using lemongrass distillates (the samples were not compared with ice cream without additives)	Sensory quality: ice creams prepared using lemongrass distillates were rated higher than those with added powder (the samples were not compared with ice cream without additives)	[207]
Curry leaf	Ice cream	Distillate (2.50% and 3.50%) and Leaf powder (0.70 and 0.75%)	Ice creams prepared using curry leaf powder had significantly higher fat, protein, carbohydrate and total solids content when compared to ice creams prepared using curry leaf distillates (the samples were not compared with ice cream without additives)	Sensory quality: ice creams prepared using curry leaf distillates were rated higher than those with added powder (the samples were not compared with ice cream without additives)	[207]
Ginger	Ice cream	Ginger juice, ginger paste and ginger syrup (5%)	Decrease in total solids, total soluble solids, fat content, and an increase in ash content increase in antioxidant activity (RP) antimicrobial properties	Increased the first dripping time Textural Properties: higher values for all textural properties (hardness, springiness, cohesiveness, gumminess and chewiness) Sensory quality: Ice creams with the addition of different forms of ginger were rated higher in taste, texture, aroma and overall acceptability (most preferred sample with syrup)	[194]
Tulsi, Ginger, Clove	Ice cream	Tulsi paste (2.5%), Ginger juice (2.0%) and Clove extract (4.0%)	Decrease in total solids, carbohydrate content, and an increase in fat, protein, and ash content	Reduction of meltdown time Specific gravity—no changes	[195]
*Psydrax umbellata*,	Herbal candy	Leaf extract (5 g)	Minor increases in protein, ash, fibre, vitamin C, total minerals, total phenolics content Increase in antioxidant activity (DPPH radical scavenging capacity)	Sensory quality—without changes in appearance, taste, texture, overall acceptance, but slightly lower in odour score	[182]

Confectionery products can be an attractive form of functional food if they are appropriately enriched with bioactive ingredients, such as herbs or spice. Their introduction requires technological, sensory and legal aspects to be taken into account. But it does also offer the opportunity to create innovative, attractive and health-promoting products that meet the needs of today’s consumers.

## 6. Technological Aspects, Quality Control and Safety of Functional Confectionery Products

Various methods are used to introduce plant additives in the production of functional sweets. Direct addition involves introducing powdered raw materials [192,197], which is simple but carries the risk of losing volatile components and changing the texture. Plant extracts (aqueous, alcoholic and oil) [196,200] allow for precise dosing of active compounds but require protection against degradation during processing. Micro- and nanoencapsulation protect bioactive ingredients from oxidation and temperature, mask intense flavours, and increase bioavailability [169,203]. Essential oils, obtained from aromatic plants, can be incorporated to impart distinctive flavour and aroma while delivering bioactive compounds with antioxidant and antimicrobial properties, though their volatility and sensitivity should be considered during processing [168,202,203].

Preparation processes for confectionery with spices/herbs addition depends on the produced form of product. In the case of hard candies, it is crucial to add ingredients at the end of cooking or to use encapsulated forms to reduce thermal losses [209]. The production of jellybeans uses milder processing conditions, which are conducive to the preservation of bioactive compounds. To ensure even distribution of additives and the right texture, it is necessary to adjust the concentration of gelling hydrocolloids to the properties of the extract [210,211,212]. In the case of baked goods, prolonged heat treatment at high temperatures is the main factor degrading bioactive ingredients; therefore, it is necessary to optimise baking parameters to limit the degradation of active ingredients [213].

Monitoring changes in the content of bioactive compounds and antioxidant activity during storage allows the maintenance of product functionality to be assessed. The addition of herbs can extend oxidative stability by delaying fat rancidity [213,214,215]. Precise determination of key bioactive substances in the finished product is necessary to confirm compliance with declarations. Product analysis at various stages of production allows the assessment of losses of active compounds and the formation of undesirable by-products. Quality control also includes the assessment of the impact of additives on taste, smell and texture, which determines consumer acceptance [216].

Standardisation of plant extracts allows for the control of both desirable and potentially toxic compounds [217]. Raw materials and end products must be free of pathogens and meet microbiological safety requirements. Herbs and spices can accumulate heavy metals or contain pesticide residues, so monitoring their levels is required [218,219].

## 7. Conclusions and Directions for Further Research

The use of herbs and spices as functional additives in the food industry, including the confectionery sector, is a promising strategy for increasing the nutritional and health-promoting value of foods. Herbs and spices are rich sources of bioactive compounds such as polyphenols, essential oils, saponins and tannins. Those compounds exhibit a range of biological properties from antioxidant and anti-inflammatory to antimicrobial and immunomodulatory effects. Their addition can improve the health profile of the product and affect sensory characteristics and microbiological stability.

In the context of confectionery products, the use of herbs and spices can contribute to the creation of a new generation of functional confectionery that meets the needs of today’s consumers who are looking for foods that are tasty but also beneficial to health. Examples of successful applications include the enrichment of chocolates, biscuits, cake, muffins and ice creams with various forms of herbs and spices, like powder, extracts, distillates and essential oils (Table 2). At the same time, research shows that appropriate dosage and processing technology are crucial to maintaining the biological activity and sensory acceptability of products.

Despite the numerous benefits, there are significant challenges associated with the use of herbs and spices in food technology. These include the thermal and oxidative instability of bioactive substances, the potential for interactions with other ingredients in the food matrix, and legal restrictions on the use of health claims. It is necessary to further develop encapsulation technologies and methods for the protection of active ingredients, as well as to conduct in vivo studies to unambiguously assess the bioavailability and bioactivity of herbs in confectionery products.

Future research should focus on standardising herbal and spice raw materials in terms of their bioactive compound content, which is key to maintaining the declared functional properties of confectionery products. It is equally important to optimise processing methods in order to minimise the loss of active substances during production and storage. At the same time, innovative confectionery matrices should be developed to protect and control the release of functional ingredients, which will increase their stability and effectiveness. Another important area of research is the analysis of bioavailability and the impact of long-term consumption of herbs and spices in confectionery products on the human body, which will allow for scientific confirmation of health claims. Last but not least, research on consumer acceptance in different demographic groups is important as it will provide insight into the perception of the health benefits and sensory qualities of such products.

An integrated approach combining knowledge from food technology, plant chemistry, dietetics and consumer science is necessary to fully exploit the potential of herbs and spices in the creation of functional confectionery products that not only satisfy taste needs but also support public health.

## Figures and Tables

**Table 1:** Bioactive compounds in selected herbs and spices and their health-promoting properties.

Herb/Spice	Main Bioactive Compounds	Main Health-Promoting Properties	References
Sage (*Salvia officinalis* L.)	Rosmarinic acid, carnosol, luteolin, thujone	Antioxidant, Antimicrobial, Anti-inflammatory	[20,21,22,23]
Rosemary (*Rosmarinus officinalis* L.)	Carnosic acid, Rosmarinic acid, Eugenol	Antioxidant, Antimicrobial, Neuroprotective	[24,25,26,27]
Peppermint (*Mentha piperita* L.)	Menthol, Menthone, Hesperidin	Antimicrobial, Anti-inflammatory, Digestive	[28,29,30]
Thyme (*Thymus vulgaris* L.)	Thymol, Carvacrol, Flavonoids	Antimicrobial, Anti-inflammatory, Antioxidant	[31,32,33,34,35,36]
Lavender (*Lavandula angustifolia* L.)	Linalool, Linalyl acetate	Sedative, Anxiolytic, Antimicrobial	[37,38,39]
Turmeric (*Curcuma longa* L.)	Curcumin, Demethoxycurcumin	Anti-inflammatory, Antioxidant, Antimicrobial	[40,41,42,43,44,45,46]
Ginger (*Zingiber officinale* L.)	Gingerol, Shogaol, Paradol	Anti-inflammatory, Antioxidant, Immunomodulatory	[47,48,49,50,51,52]
Tulasi (*Ocimum tenuiflorum* L.)	Eugenol, Ursolic acid, Rosmarinic acid	Antimicrobial, Anti-inflammatory, Anticancer	[53,54,55,56,57]
Chamomile (*Matricaria recutita* L.)	Apigenin, Chamazulene, α-Bisabolol	Antioxidant, Antimicrobial, Anti-inflammatory	[58,59,60,61,62]
Common basil (*Ocimum basilicum* L.)	Linalool, Eugenol, Methyl chavicol	Antioxidant, Antimicrobial, Anti-inflammatory	[63,64,65]
Garlic (*Allium sativum* L.)	Allicin, Ajoene, S-allylcysteine	Antimicrobial, Antioxidant, Immunomodulatory	[66,67,68,69,70,71]
Lemon balm (*Melissa officinalis* L.)	Rosmarinic acid, Citral, Flavonoids	Antioxidant, Antimicrobial, Gastroprotective	[72,73,74,75,76]
Fenugreek (*Trigonella foenum-graecum* L.)	Trigonelline, Saponins, Flavonoids	Anti-inflammatory, Antioxidant, Hypoglycemic	[77,78,79,80,81]
Liquorice (*Glycyrrhiza glabra* L.)	Glycyrrhizin, Glabridin, Liquiritin	Anti-inflammatory, Antiviral, Hepatoprotective	[82,83,84,85,86,87]
Nigella (*Nigella sativa* L.)	Thymoquinone, Nigellone	Antioxidant, Antimicrobial, Immunomodulatory	[88,89,90,91,92,93,94,95]
Oregano (*Origanum vulgare* L.)	Carvacrol, Thymol, Rosmarinic acid	Antioxidant, Antimicrobial, Anti-inflammatory	[96,97,98,99]
Purple Coneflower (*Echinacea purpurea* L.)	Cichoric acid, Alkamides, Polysaccharides	Immunomodulatory, Antioxidant, Antiviral	[100,101,102,103,104]

## Data Availability

Data sharing not applicable.
